# The Nationalisation of the Profession

**Published:** 1910-10-15

**Authors:** 


### The Hospital

A Journal of

The Medical Sciences and Hospital Administration.

New Series. No. 192, Vol. VIII. [No. 1264, Vol. XLIX.] SATURDAY, OCTOBER 15, 1910.

### THE NATIONALISATION OF THE PROFESSION.

Since the Royal Commission on the Poor Law published its dual report we have been treated to a variety of opinions and a diversity of schemes which, to say the least, are extremely puzzling to the man or woman who honestly wishes to make up his or her mind for or against one or other of the summary of suggestions proposed by the Commissioners. The harvest of discussion has not yet been reaped. Probably it will not be gathered in for several years, and even then it is only reasonable to expect that we shall be confronted with an aftermath as gigantic as the original gathering. But one thing at least is clear, and that is that the signatories of the Minority Report have lost no time in putting their views, with the fullest explanatory comments, before the public at large. Their original report was full enough, but the added facts gathered from all sources have greatly enriched the material which they possess for propaganda purposes, and it is really no fault of theirs if the profession does not yet realise what their suggestions mean.

One of the latest additions to the already voluminous literature on the subject is Mr. and Mrs. Webb's conjoint commentary on the medical aspects of these proposals,* in which the matter is dealt with in a manner that for lucidity and clearness it would be hard to beat. The first five chapters of this book we can afford to pass over. They are, in summary, a *précis* of the arguments against the existing system of medical relief. Whoever has read and digested the minutes of evidence of the Commission will be in a position to consider the relative merit of this testimony, often conflicting, but always furnishing food for reflection and discussion. It is merely in the interpretation and, above all, in the application of the conclusions to be drawn from such evidence that there is justification for the most profound difference of opinion. And that difference must remain so long as there is reasonable ground for variously estimating the relative value of the evidence. Probably the consensus of calm opinion will be that there is something rotten in the present system. We are all agreed that the established order of things is not perfect. But the question still is, In what manner is it to be changed if change is deemed advisable?

The authors are at hand with a solution of this question, and in the concluding chapters they outline a scheme which needs serious attention and thoughtful consideration. They demand the creation of a unified medical service "in which the medical services of the Poor Law and the Public Health authorities would be merged." In their opinion this will overcome the overlapping which they assure us is present in an appalling degree and which, to a large extent, stultifies much of the good that could be achieved under the present system. They argue that the establishment of such a unified medical service on public health lines—which is a circumlocutory way of paraphrasing the nationalisation of the profession—does not necessarily involve the gratuitous provision of medical treatment to all applicants, and suggest provision for all possible contingencies in the shape of definite rules of chargeability to be drawn up and sanctioned by the Legislature. They refuse to accept Dr. Downes' proposal to put the public health hospitals directly under the Poor Law authority, but plead for the adoption of what is after all the scheme outlined by the majority of expert witnesses called before the Commission, the delegation to the Public Health Department of practically all cases of tuberculosis, cancer, and rheumatism—in other words, the transference of all the infirmary cases to the care of the Public Health authority. It must be remembered that this latter scheme has the support of nearly every medical practitioner who has conscientiously and carefully studied the subject, but that such support is not unqualified. There is, in the first place, the question of the fate of the private practitioners to be considered. We do not imagine for a moment that any professional man will be selfish enough to put his private interests in the scale against national efficiency and communal sanitation. But we cannot conceive that the profession is likely to be cajoled into accepting an arrangement which, however excellent it may appear to be when formulated, may ultimately be found to have its drawbacks in practice. Mr. and Mrs. Webb have, in this book at least, done no more to convince us of the practicability of the outlined scheme than the expert witnesses before the Commission, whose views they have adopted, and we do not think that professional men will be inclined to accept as final the summarised conclusions of two lay people, however capable these latter may be, in the face of adverse professional opinion. Does such adverse opinion exist? That is the question, and it is not an easy one to decide. There have been singularly hard and biased verdicts passed on the medical aspects of the Minority Report's recommendations, but these lack impressiveness because the judges have been partisans or woefully ignorant of facts. What is wanted is a free and general discussion of principles. We believe the formation of the recent Medical Committee on the Poor Law is likely to do much towards furthering and encouraging a frank expression of professional opinion on the whole matter. Such an expression is imperatively necessary, and until it is enunciated no one can honestly say what the profession, as a whole, thinks of the proposals, and how far it is inclined to be nationalised. And without its will and co-operation no profession, least of all that of medicine, can be summarily turned into a department of State.

* *The State and the Doctor.* By S. and B. Webb. Longmans, Green and Co. 6s. net.

